# Adjuvant 5‐Fluorouracil/leucovorin, capecitabine, and oxaliplatin‐related regimens for stage II/III colon cancer patients 66 years or older

**DOI:** 10.1002/cam4.5078

**Published:** 2022-10-13

**Authors:** Emily Jones, Zhigang Duan, Thinh T. Nguyen, Sharon H. Giordano, Hui Zhao

**Affiliations:** ^1^ Department of Biostatistics The University of Texas School of Public Health at Houston Houston Texas USA; ^2^ Department of Health Services Research The University of Texas MD Anderson Cancer Center Houston Texas USA; ^3^ Institute for Clinical and Translational Research, Baylor College of Medicine Houston Texas USA; ^4^ Department of Breast Medical Oncology The University of Texas MD Anderson Cancer Center Houston Texas USA

## Abstract

Adjuvant chemotherapy of leucovorin‐modulated 5‐fluorouracil (5‐FU/LV), capecitabine, and adding oxaliplatin to 5‐FU/LV or capecitabine (FLOX/OX) have been standard regimens for high‐risk stage II or III colon cancer (CC). We aimed to evaluate their patterns of use, association with survival, and rate of emergency room visit (ER) or hospitalization during the treatment period. High‐risk stage II or III patients aged >65 years diagnosed between 2007 and 2015, underwent colectomy, and received any of these three regimens were selected from SEER and Texas Cancer Registry (TC) linked with Medicare data. Chi‐square test, Kaplan–Meier survival curves, Cox regression, and logistic regression were used in data analysis. A total of 5621 (1080 stage II and 4541 stage III) patients with median age of 72 years were included in this study. For stage II, 24.4% used 5‐FU/LV, 31.2% used capecitabine, and 44.4% used FLOX/OX; the respective numbers for stage III were 13.8%, 17.9%, and 68.3%. Patients aged <70 years, not in the West region, not in Medicare state‐buy‐in program, and with no comorbidity were more likely to use FLOX/OX. FLOX/OX was associated with improved overall survival (OS) in stage II and III patients and improved cancer‐specific survival in stage III patients compared with 5‐FU/LV. The survival benefit of FLOX/OX was sustained in stage III patients aged ≥70 years. Capecitabine had the lowest ER/hospitalization rate with 19.2% in stage II and 28.9% in III. The use of FLOX/OX was associated with improved survival compared with 5‐FU/LV among CC patients. Capecitabine was associated with the lowest ER/hospitalization rate.

## Graphical abstract

FLOX/OX treatment was associated with improved overall survival (OS) and improved cancer specific survival in stage III patients compared with 5‐FU/LV and the survival benefit of FLOX/OX was sustained in stage III patients aged 70 years. Capecitabine had the lowest emergency room visit/hospitalization among the three regimens with 19.2% in stage II and 28.9% in stage III patients.

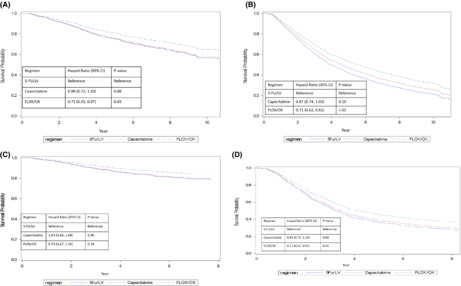

## 1 INTRODUCTION

Colon cancer is the third most commonly diagnosed cancer in the United States for both males and females. The probability of developing colon cancer increases with age; men and women aged 70 and older have a 1 in 32 or 1 in 34 chance of colon cancer development, respectively. From 2010 to 2016, 35% of colon cancer diagnoses were considered regional (stage II) and 22% were considered distant (stage III+). Patients diagnosed with stage II colon cancer had relative 5‐year survival around 72%, while stage III+ patients had the lowest chance of survival at 14%.^1^

The National Comprehensive Cancer Network (NCCN) recommends for high‐risk stage II and III colon cancer patients to receive chemotherapy after colectomy.^2^ There are currently multiple combination adjuvant chemotherapy regimens available for colon cancer treatment using different combinations or amounts of the drugs fluorouracil, leucovorin, oxaliplatin, and capecitabine. Some regimens include: 5‐FU/LV [fluorouracil, leucovorin], FLOX [fluorouracil, leucovorin, oxaliplatin], capecitabine alone or CAPOX [capecitabine with oxaliplatin].^2^, ^3^ The most commonly used regimens and those recommended by the NCCN are FLOX and CAPOX.^2^, ^4^ These regimens have been standard practice for around 15 years.^3^

Prior to the mid‐1980s, 5‐FU/LV was the standard practice of treatment; however, rapid pharmaceutical advances led to the creation of new regimens.^4^ Capecitabine was approved by the FDA in 1998 for treatment of these patients, with oxaliplatin approved in 2004.^2^, ^5^ Oxaliplatin was combined with 5‐FU/LV to create the FLOX regimen,^2^, ^5^ and also with capecitabine to create the CAPOX regimen in 2005.^3^ According to the NCCN, low‐risk stage III colon cancer patients should receive 3 months of CAPOX while high‐risk patients should receive 6 months of either CAPOX or FLOX.^2^, ^3^ Studies have shown that CAPOX and FLOX have similar survival outcomes.^6^

One of the largest trials that suggested a survival benefit of adjuvant chemotherapy in patients with stage II colon cancer was the QUASAR (Quick and Simple and Reliable Study). The trial reported that the relative risk of death from any cause with chemotherapy versus observation alone was 0.82 (95% CI 0.70–0.95) and the relative risk of recurrence with chemotherapy versus observation alone was 0.78 (95% CI 0.67–0.91). This was interpreted to state that chemotherapy with fluorouracil and folinic acid improved the survival of patients, even if only by a small percentage.^7^

The MOSAIC and NSABP C‐07 trials established the benefit of the addition of oxaliplatin in adjuvant therapy for stage II and III disease, especially in patients under 70 years of age.^8^, ^9^ More recent studies have focused on patients using Medicare who are often over 65 years of age. These studies have concluded that administration of adjuvant chemotherapy for stage II and III colon cancer decreases with advancing age, and adjuvant chemotherapy is associated with a decreased risk of mortality in stage III patients more so than stage II patients, consistent across all age groups except 90 or above.^10^, ^11^ Many studies still question if adjuvant chemotherapy for stage II diagnoses provides a substantial improvement in overall survival of elderly patients.^10^, ^11^

Many of the above studies investigated adjuvant chemotherapy treatment patterns before 2005 when 5‐FU was the main option, or they examined survival after adjuvant chemotherapy without stratifying by chemotherapy regimen received. Few studies have evaluated treatment adverse events such as emergency room (ER) visits or hospitalizations while patients are receiving chemotherapy. Since treatment options for colon cancer have advanced and expanded since 2005, determining patterns of adjuvant chemotherapy regimen use and associated overall survival rates is of utmost importance. The objective of this study is to explore treatment patterns and survival trends by adjuvant chemotherapy regimen and ER/hospitalization during chemotherapy treatment period in the SEER and Texas Cancer Regis‐Medicare population (Medicare beneficiaries aged 65+) in more recent years.

## 2 STUDY MATERIAL AND METHODS

We used SEER‐Medicare and TCR‐Medicare data that covered cancer diagnosis between 2007–2015 and 2009–2015, respectively (TCR‐Medicare data were available to us since 2009). SEER is a population‐based cancer registry from 17 SEER areas. TCR is one of the largest cancer registries in the United States and is one of the 12 state registries funded by both the SEER program and Centers for Disease Control and Prevention's National Program of Cancer Registries. It includes patients' sociodemographic information such as age, gender, marital status, state‐buy‐in and Medicare enrollment, education and income level, cancer diagnosis and stage, histology type, AJCC tumor stage, tumor grade, and cancer treatment information such as vital status, surgical removal, and number of lymph nodes. Medicare claims include information on cancer treatment type (chemotherapy, type of surgery, etc.), ER visits, hospitalizations, and corresponding dates. We used the International Classification of Diseases 9th or 10th Revision (ICD‐9 or ICD‐10) procedure codes and Healthcare Common Procedure Coding System (HCPCS) codes (Appendix Table A1) to identify patients' chemotherapy use, cancer‐directed surgery, and ER visits or hospitalizations.

**FIGURE 1:**
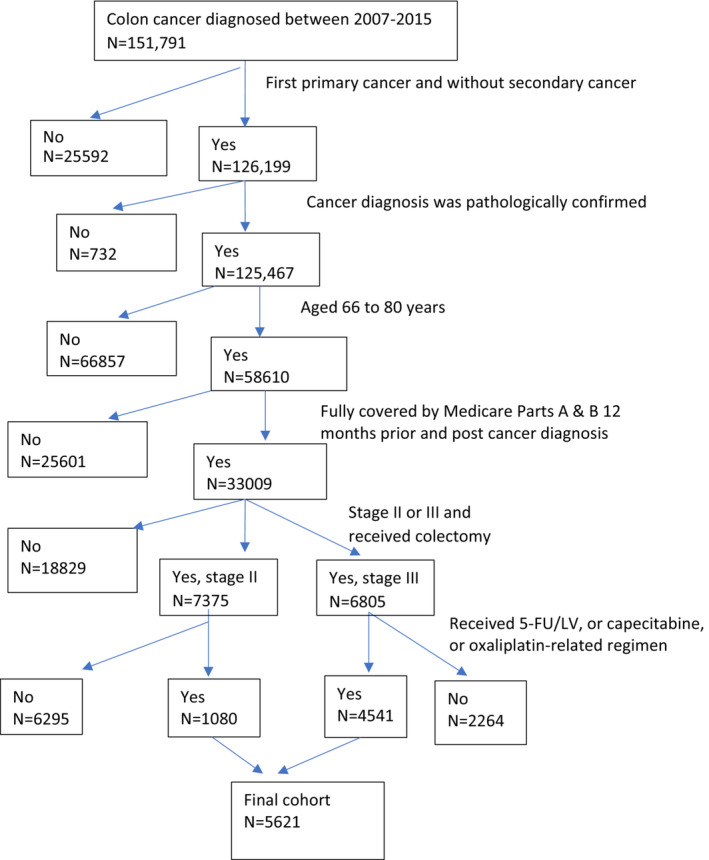
Cohort selection of stage II or III colon cancer patients diagnosed between 2007 and 2015 in the SEER‐ and TCR‐Medicare.

We used the following criteria for cohort selection (Figure 1 displays the cohort selection process). (1) Diagnosed with colon adenocarcinoma between 2007–2015 for SEER‐Medicare data and 2009–2015 for TCR‐Medicare data. We chose this study period as Medicare data began including prescription drugs in 2007, and our current data use agreement with SEER‐Medicare and TCR‐Medicare supported this time window. (2) The colon cancer was first primary cancer stage II (T3 or T4, N0, M0) or stage III (any T, N1 or N2, M0) and without secondary cancer. (3) The cancer diagnosis was biologically and pathologically confirmed and was not by autopsy or death certificate only. (4) Patients were aged 66 to 80 years. (5) Patients had continuous Medicare enrollment 1 year prior and after cancer diagnosis. (6) Patients received colectomy within 6 months since cancer diagnosis and initiated chemotherapy within 4 months since cancer diagnosis. We further selected patients whose chemotherapy regimen included 5FU/LV, capecitabine, or FLOX/OX.

We grouped chemotherapy regimens in three categories: 5‐FU/LV, capecitabine, and FLOX/OX. The time window used for defining regimen types was from first until last chemotherapy claim. If patients' first chemotherapy date had one of the aforementioned regimens, the patient was defined as receiving that regimen. During the chemotherapy treatment period, if there was a gap of ≥90 days between two chemotherapy claims, we censored patients' chemotherapy treatment before the gap. The gap may be an indication of ineffective treatment, treatment noncompliance, disease progression, or other issues. In this study, we only evaluated patients' initial chemotherapy regimen type. We computed patients' chemotherapy treatment duration as months between first and last chemotherapy claim date of the initial chemotherapy regimen type. Based on the treatment duration, we grouped patients who received chemotherapy into 1–3, 3–6, and >6 months. Other outcome variables in our study were overall survival (OS) and cancer cause‐specific survival (CSS). Since chemotherapy treatment is associated with toxicities, we used ER visit and hospitalization during the chemotherapy period as a surrogate for severe adverse events or treatment complications related to chemotherapy. Covariates in our study were patients' sociodemographic factors and tumor characteristics.

We used frequency and percentage to describe types of regimens used in stage II and III patients. We reported median and interquartile range for treatment duration and survival time by cancer stage and regimen type. We used Chi‐square tests to compare associations between covariates and different regimens. Cochran–Mantel–Haenszel tests were used to evaluate the yearly trend by regimen. Multivariable Cox‐regression models were used to evaluate risk of death for different regimens, and Kaplan–Meier survival curves based on multivariable Cox regression models were used to compute overall and cancer cause‐specific survival probability.^12^, ^13^ To further control patients' baseline differences in the choice of regimens, we used logistic regression analysis to estimate the propensity of regimen by including patients' baseline conditions such as year of cancer diagnosis, gender, age, race, marital status, geographic region, urban and rural area, enrolled in Medicare state‐buy‐in program, census track level of education and median household income, Charlson comorbidity index, tumor stage, tumor grade, and number of lymph nodes removed. Then, we used multivariable Cox regression model with propensity score quintile stratification to estimate the hazard ratio for capecitabine, and FLOX/OX compared with 5Fu/LV. This approach showed to eliminate about 90% of the bias due to confounders.^14^ We estimated the ER and hospitalization rates by regimen type and used logistic regression models to report odds ratios. We also reported 95% confidence intervals (CI) for survival probabilities, hazard ratios (HR), and odds ratios (OR). All statistical tests were two‐sided with statistical significance level at 0.05. This study was using de‐identified patients' cancer diagnosis and treatment information from the SEER and TCR data linked with Medicare. The study was exempted by the Institutional Review Board at The University of Texas MD Anderson Cancer Center. Data analysis was performed using SAS Enterprise Guide 7.1 (SAS Institute, Cary, NC).

## 3 RESULTS

**FIGURE A1:**
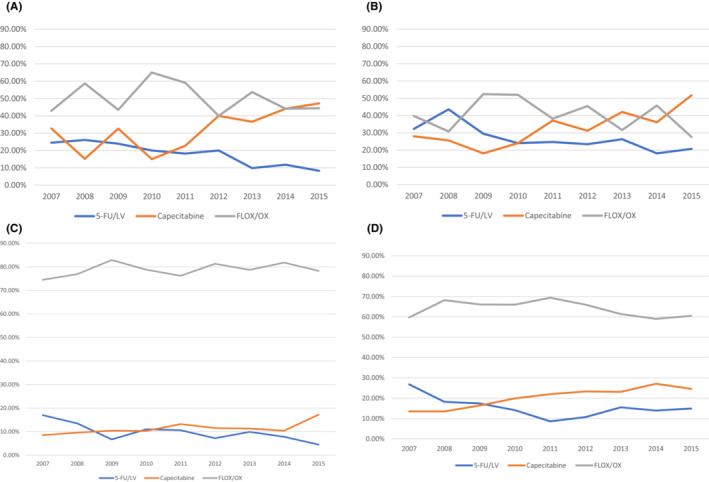
(A) Chemotherapy regimen distribution for stage II colon cancer aged 66–69 years between 2007 and 2015. (B) Chemotherapy regimen distribution for stage II colon cancer aged ≥70 years between 2007 and 2015. (C) Chemotherapy regimen distribution for stage III colon cancer aged 66–69 years between 2007 and 2015. (D) Chemotherapy regimen distribution for stage III colon cancer aged ≥70 years between 2007 and 2015. 5‐FU/LV is leucovorin (LV)‐modulated 5‐fluorouracil. FLOX/OX is oxaliplatin added to 5‐FU/LV or capecitabine. The 95% CI is 95% confidence interval.

There were 1080 stage II and 4541 stage III patients; 44.4% and 68.2% used FLOX/OX, 31.2% and 17.8% used capecitabine, and 24.3% and 13.7% used 5‐FU/LV, respectively. The median treatment duration for these regimens was around 5 months with IQR [1.7–6.4] months. (Appendix Figures A1A–D).

### 3.1 Factors associated with type of chemotherapy regimen

Based on Chi‐square tests, we found several factors associated with type of regimen used for stage II/III patients. For both stage II and III patients, the use of 5‐FU/LV decreased while capecitabine increased over the study years (Table 1). The use of FLOX/OX increased from 2007 to 2011 but began decreasing in 2012. This was mainly observed in patients ≥70 years (Appendix Figure A1A–D). Besides age, other factors such as not living in West SEER regions, not being enrolled in state‐buy‐in programs, and having no comorbidities were associated with greater oxaliplatin‐related regimen use. For stage II patients, having T4 stage tumors or surgical removal of <12 lymph nodes were associated with higher FLOX/OX use. For stage III patients, sociodemographic factors including being married, living in high education, income, or metro areas, or with high tumor grade were related to higher FLOX/OX use.

**TABLE 1:** Distribution of chemotherapy regimens by cancer stage and patients' characteristics

		Stage II					Stage III				
			5‐FU/LV	Capecitabine	FLOX/OX			5‐FU/LV	Capecitabine	FLOX/OX	
		Total N	(%)	(%)	(%)	p	Total N	(%)	(%)	(%)	p
Year of diagnosis	2007–2009	417	**31.1**	**24.7**	**44.1**	**≤0.01**	1473	14	13.1	68.5	**≤0.01**
	2010–2012	365	22.4	29.3	48.2		1537	10.7	**18.8**	70.3	
	2013–2015	298	17.1	42.6	40.2		1531	12.5	21.4	65.9	
Age	66–69	376	**18.6**	31.1	50.2	**<0.01**	1297	9.5	**11.4**	78.9	**<0.01**
	70–80	704	27.4	31.2	41.3		3244	15.4	20.4	64.0	
Gender	Female	571	24.8	31.6	43.4	0.78	2330	13.9	18.6	67.4	0.38
	Male	509	23.7	30.6	45.5		2211	13.6	17.1	69.1	
Race	White	829	24.4	32.9	42.5	0.29	3498	13.7	17.3	68.8	**<0.01**
	Black	74	27.0	25.6	47.2		383	**15.6**	14.8	**69.4**	
	Hispanic	114	22.8	23.6	53.5		372	**13.4**	**19.0**	67.4	
	Other/Unknown	63	22.2	28.5	49.2		288	13.1	26.3	**60.4**	
Region	Northeast	185	32.4	**26.4**	41.0	**<0.01**	724	16.1	**12.1**	71.6	**<0.01**
	Midwest	112	28.5	23.2	48.2		479	**18.7**	13.9	67.2	
	South	426	22.0	**28.4**	**49.5**		1929	11.1	18.5	70.3	
	West	357	**21.5**	**39.4**	**38.9**		1409	**14.4**	**21.3**	**64.1**	
AJCC T	T1	NA	NA		NA		172	**9.3**	**23.8**	66.8	**0.02**
	T2	NA	NA	NA	NA		395	**10.8**	20.2	68.8	
	T3	742	**27.0**	**31.8**	**41.1**	**<0.01**	3092	**13.7**	17.8	**68.4**	
	T4	338	**18.3**	**29.8**	**51.7**		882	**16.2**	15.7	68.0	
AJCC N	N1	NA	NA	NA	NA	NA	3006	13.8	19.4	66.6	**<0.01**
	N2	NA		NA	NA		1535	13.6	14.8	**71.5**	
Grade	Well differentiate	73	26.0	34.2	39.7	0.09	218	10.5	19.7	69.7	**0.04**
	Moderately differentiate	746	26.2	30.8	42.8		2992	**13.3**	**18.7**	67.9	
	Poorly/un‐differentiate	261	18.3	31.4	50.1		1331	15.4	15.7	68.8	
Surgical remove lymph nodes	<12 LN removed	216	**30.0**	**22.6**	**47.2**	**<0.01**	605	15.3	17.8	66.7	0.50
	≥12 LN removed	864	22.9	**33.3**	43.7		3936	13.6	17.8	68.4	
Marital status	Married	589	24.2	29.0	46.6	0.18	2541	13.6	**16.2**	**70.0**	**<0.01**
	Not married	491	24.4	33.8	41.7		2000	**14.0**	**19.9**	**65.9**	
Enrolled in Medicare state‐buy‐in	No	874	25.1	**29.0**	**45.7**	**<0.01**	3777	**13.5**	**16.0**	**70.4**	**<0.01**
	Yes	206	20.8	40.2	**38.8**		764	**15.1**	**27.1**	**57.6**	
Urban/rural	Big Metro	547	23.4	31.2	45.3	0.76	2236	**12.0**	**18.3**	**69.5**	**0.02**
	Metro	307	25.7	32.2	42.0		1403	**15.4**	**16.3**	**68.1**	
	Urban	187	23.5	31.5	44.9		791	**15.1**	**19.3**	**65.4**	
	Rural	39	<35.0^a^	<25.0^a^	48.7		111	**17.8**	**16.9**	**65.1**	
Census tract median income	Q1 $0–36,811	309	26.7	31.2	41.9	0.58	1172	**13.7**	**21.0**	**65.1**	**<0.01**
	Q2 $36812–51,033	260	24.9	29.8	45.2		1184	**15.7**	**16.3**	**67.9**	
	Q3 $51034–71,033	260	24.2	31.7	44.0		1125	**14.3**	**17.0**	**68.6**	
	Q4 ≥ $71,034	251	20.3	32.0	47.6		1060	**11.1**	**17.0**	**71.8**	
Census tract non high school graduate percent	Q1 ≥ 22.53%	336	6.2	31.0	42.7	0.73	1261	**13.1**	**21.0**	**65.8**	**<0.01**
	Q2 12.96–22.52%	261	3.8	31.5	44.6		1136	**14.8**	**18.1**	**66.9**	
	Q3 7.06–12.95%	252	23.0	27.6	49.2		1071	**15.3**	**15.0**	**69.6**	
	Q4 0.0–7.05%	231	23.9	34.6	41.4		1073	**11.8**	**16.8**	**71.2**	
Charlson index	0	567	**22.0**	**30.1**	**47.7**	**<0.01**	2332	**11.2**	**15.9**	**72.8**	**<0.01**
	1	293	**25.9**	**27.9**	**46.0**		1179	**15.7**	**16.9**	**67.3**	
	2+	220	**28.1**	38.1	33.6		1030	**17.4**	**23.4**	**59.0**	
Chemotherapy duration	<90 days	256	**24.2**	42.1	33.5	**<0.01**	817	**14.3**	**31.7**	**53.9**	**<0.01**
	90–180 days	597	**22.2**	29.8	47.9		2679	**11.4**	**16.6**	**71.9**	
	181+ days	227	**29.9**	22.4	47.5		1045	**19.6**	**10.2**	**70.1**	

Abbreviations: AJCC, American Joint Committee on Cancer; LN, lymph nodes; Q, quartile; NA, not applicable.

a The actual value of the percentage in a cell and its adjacent cell in this table with number of patients less than 11 was not reported so no cell with <11 patients can be derived from the table. This is to protect patients' confidentiality.

Bold indicates all the percentages in associated with a significant *p*‐values.

### 3.2 Association between regimens and patients' survival

**FIGURE 2:**
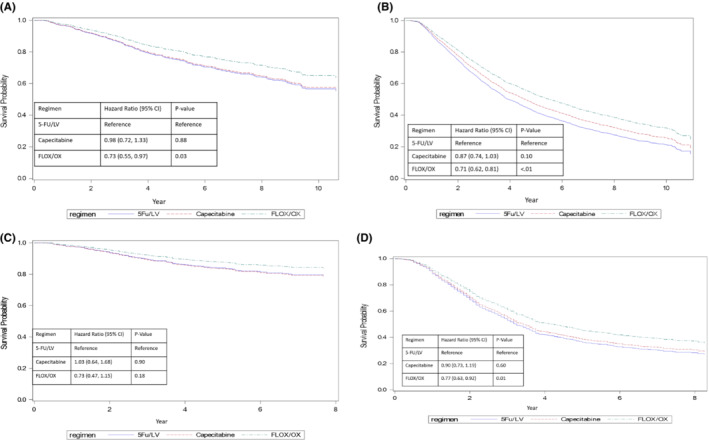
(A) Overall survival curves generated based on multivariable Cox regression model for stage II colon cancer patients by regimen. (B) Overall survival curves generated based on multivariable Cox regression model for stage III colon cancer patients by regimen. (C) Cancer‐specific survival curves generated based on multivariable Cox regression model for stage II colon cancer patients by regimen. (D) Cancer‐specific survival curves generated based on multivariable Cox regression model for stage III colon cancer patients by regimen. 5‐FU/LV is leucovorin (LV)‐modulated 5‐fluorouracil. FLOX/OX is oxaliplatin added to 5‐FU/LV or capecitabine. The 95% CI is 95% confidence interval.

Compared with 5‐FU/LV, FLOX/OX was associated with improved OS in stage II and III patients, and improved CSS for stage III patients (Figure 2A–D, Table 2A,B). In stage II and III patients, the adjusted 5‐year OS rates were 80.8% and 53.3% for FLOX/OX and 75.0% and 42.3% for 5‐FU/LV, respectively (Stage II HR = 0.73, 95% CI (0.55–0.97), Stage III HR = 0.71, 95% CI (0.62–0.81)). For stage III patients, the 5‐year CSS for patients using FLOX/OX was 46.6% vs. 37.4% for 5‐FU/LV (HR = 0.77, 95% CI [0.63–0.92]). Compared with 5‐FU/LV, capecitabine had similar OS and CSS for both stage II and III patients. Covariates associated with better OS for stage II patients include: diagnosed between 2010 and 2012, age <70 years, being female, married, living in lowest median income areas, no comorbidity, T3 stage tumor, and receiving chemotherapy for ≥3 months. Factors associated with better CSS in stage II patients include: diagnosed between 2010 and 2012, age <70 years, receiving chemotherapy for 3–6 months, T3 stage tumor, and surgical removal of >12 lymph nodes. In stage III patients, covariates associated with better OS include: being female, living in rural areas or areas with percent of not attaining a high school degree of 13%–23%, no comorbidity, T1 or N1 stage or well‐differentiated tumor, receiving chemotherapy treatment ≥3 months, and surgical removal of >12 lymph nodes. For CSS in stage III patients, all factors were similar to OS in stage III patients except that sex and age were not associated.

**TABLE 2:** Multivariable Cox regression models of overall survival and cancer‐specific survival for stage II colon cancer patients

Regimens		Overall survival		Cancer‐specific survival	
		HR (95% CI)	*p*	HR (95% CI)	*p*
(A)					
Regimen	5‐FU/LV (ref)				
	Capecitabine	0.98 (0.72, 1.33)	0.88	1.03 (0.64, 1.68)	0.90
	FLOX/OX	**0.73 (0.55, 0.97)**	**0.03**	0.73 (0.47, 1.15)	0.18
Year of diagnosis	2007–2009 (ref)				
	2010–2012	0.78 (0.59, 1.02)	0.07	**0.60 (0.39, 0.92)**	**0.02**
	2013–2015	0.84 (0.58, 1.21)	0.34	0.92 (0.47, 1.80)	0.80
Gender	Female (ref)				
	Male	**1.32 (1.04, 1.67)**	**0.02**	1.02 (0.70, 1.47)	0.93
Age	66–69 (ref)				
	70–80	**1.66 (1.26, 2.17)**	**<0.01**	**2.34 (1.46, 3.77)**	**<0.01**
Race	White (ref)				
	Black	1.22 (0.78, 1.93)	0.38	1.32 (0.63, 2.79)	0.46
	Hispanic	0.92 (0.59, 1.42)	0.70	1.42 (0.77, 2.60)	0.26
	Other/Unknown	0.54 (0.29, 1.03)	0.06	0.94 (0.42, 2.11)	0.89
Marital status	Married (ref)				
	Not married	**1.32 (1.04, 1.68)**	**0.03**	1.33 (0.90,1.95)	0.15
Region	Northeast (ref)				
	Midwest	1.08 (0.69, 1.69)	0.75	1.31 (0.63, 2.73)	0.48
	South	1.10 (0.76, 1.59)	0.62	1.30 (0.71, 2.40)	0.39
	West	1.08 (0.75, 1.54)	0.69	1.34 (0.75, 2.38)	0.33
Urban	Big Metro (ref)				
	Metro	1.15 (0.87, 1.51)	0.34	1.24 (0.80, 1.92)	0.34
	Urban	1.03 (0.72, 1.48)	0.88	0.95 (0.52, 1.71)	0.85
	Rural	1.47 (0.81, 2.66)	0.20	2.10 (0.91, 4.83)	0.08
Enrolled in Medicare state‐buy‐in	No (ref)				
	Yes	1.10 (0.79, 1.52)	0.58	1.35(0.82, 2.20)	0.24
Census tract non high school graduate percent	Q1 > 22.53% (ref)				
	Q2 12.96–22.52%	0.89 (0.63, 1.25)	0.49	0.81 (0.47, 1.38)	0.43
	Q3 7.06–12.95%	1.07 (0.73, 1.56)	0.74	1.03 (0.57, 1.87)	0.93
	Q4 0.0–7.05%	0.77 (0.48, 1.24)	0.28	0.66 (0.31, 1.42)	0.29
Census tract median income	Q1 $0–36,811 (ref)				
	Q2 $36812–51,033	**1.45 (1.03, 2.05)**	**0.04**	0.99 (0.57, 1.72)	0.96
	Q3 $51.34–71,033	1.21 (0.81, 1.81)	0.36	1.17 (0.64, 2.16)	0.61
	Q4 > $71,034	1.39 (0.85, 2.29)	0.19	1.25 (0.58, 2.70)	0.58
Charlson index	0 (ref)				
	1	**1.43 (1.08, 1.88)**	**0.01**	1.29 (0.85, 1.97)	0.23
	2+	**1.81 (1.35, 2.42)**	**<0.01**	1.01 (0.62, 1.65)	0.96
Chemotherapy duration	<90 days (ref)				
	90–180 days	**0.65 (0.49, 0.85)**	**<0.01**	**0.50 (0.32, 0.76)**	**<0.01**
	181+ days	**0.70 (0.50, 0.98)**	**0.04**	0.63 (0.38, 1.04)	0.07
AJCC T	T3 (ref)				
	T4	**2.15 (1.68, 2.76)**	**<0.01**	**3.41 (2.33, 4.99)**	**<0.01**
Grade	Well differentiated (ref)				
	Moderately differentiated	1.69 (0.96, 2.97)	0.07	1.09 (0.49, 2.41)	0.84
	Poorly/undifferentiated	1.49 (0.81, 2.74)	0.20	1.24 (0.53, 2.91)	0.63
LN	<12 LN removed (ref)				
	≥12 LN removed	0.87 (0.66, 1.14)	0.31	**0.63 (0.42, 0.95)**	**0.03**

		Overall survival		Cancer‐specific survival	
		HR (95% CI)	*p*	HR (95% CI)	*p*
**(B)**					
Regimen	5‐FU/LV (ref)				
	Capecitabine	0.87 (0.74, 1.03)	0.10	0.9 (0.73, 1.19)	0.60
	FLOX/OX	**0.71 (0.62, 0.81)**	**<0.01**	**0.77 (0.63, 0.92)**	**<0.01**
Diagnosis year	2007–2009 (ref)				
	2010–2012	0.90 (0.80, 1.01)	0.07	**0.83 (0.71, 0.97)**	**0.02**
	2013–2015	0.88 (0.77, 1.02)	0.08	0.84 (0.66, 1.07)	0.15
Gender	Female (ref)				
	Male	**1.29 (1.17, 1.43)**	**<0.01**	1.08 (0.93, 1.24)	0.31
Age	66–69 (ref)				
	70–80	**1.18 (1.05, 1.32)**	**<0.01**	1.09 (0.93, 1.28)	0.29
Race	White (ref)				
	Black	1.09 (0.91, 1.30)	0.35	1.14 (0.88, 1.48)	0.32
	Hispanic	1.02 (0.85, 1.23)	0.83	1.18 (0.91, 1.53)	0.21
	Other/Unknown	0.85 (0.67, 1.07)	0.15	0.93 (0.67, 1.29)	0.67
Marital status	Married (ref)				
	Not married	1.09 (0.98, 1.21)	0.12	0.99 (0.86, 1.16)	0.94
Region	Northeast (ref)				
	Midwest	1.04 (0.85, 1.27)	0.69	1.19 (0.90, 1.57)	0.21
	South	1.13 (0.97, 1.31)	0.13	1.22 (0.99, 1.52)	0.07
	West	0.92 (0.79, 1.08)	0.33	0.98 (0.78, 1.22)	0.84
Urban	Big Metro (ref)				
	Metro	1.03 (0.92, 1.15)	0.67	1.04 (0.89, 1.22)	0.61
	Urban	0.96 (0.83, 1.12)	0.62	0.97 (0.78, 1.19)	0.75
	Rural	**0.69 (0.48, 0.98)**	**0.04**	**0.52 (0.30, 0.91)**	**0.02**
Enrolled in Medicare state‐buy‐in	No (ref)				
	Yes	1.03 (0.89, 1.18)	0.72	0.91 (0.74, 1.13)	0.39
Census tract non high school graduate percent	Q1 > 22.53% (ref)				
	Q2 12.96–22.52%	**0.82 (0.71, 0.95)**	**<0.01**	**0.75 (0.61, 0.93)**	**<0.01**
	Q3 7.06–12.95%	0.92 (0.77, 1.09)	0.34	0.97 (0.76, 1.24)	0.81
	Q4 0.0–7.05%	0.84 (0.69, 1.03)	0.09	0.87 (0.66, 1.16)	0.34
Census tract median income	Q1 $0–36,811 (ref)				
	Q2 $36812–51,033	1.04 (0.90, 1.20)	0.58	1.19 (0.97, 1.46)	0.10
	Q3 $51.34–71,033	0.95 (0.80, 1.13)	0.56	1.07 (0.84, 1.38)	0.57
	Q4 > $71,034	1.00 (0.81, 1.23)	0.98	1.03 (0.76, 1.39)	0.86
Charlson index	0 (ref)				
	1	**1.21 (1.07, 1.36)**	**<0.01**	1.07 (0.90, 1.26)	0.46
	2+	**1.63 (1.45, 1.84)**	**<0.01**	**1.28 (1.07, 1.52)**	**<0.01**
Chemotherapy duration	<90 days (ref)				
	90‐180 days	**0.57 (0.50, 0.64)**	**<0.01**	**0.50 (0.42, 0.60)**	**<0.01**
	181 + days	**0.72 (0.62, 0.83)**	**<0.01**	**0.71 (0.58, 0.86)**	**<0.01**
AJCC T	T1 (ref)				
	T2	1.20 (0.81, 1.78)	0.37	0.60 (0.30, 1.19)	0.14
	T3	**1.83 (1.30, 2.58)**	**<0.01**	**1.85 (1.08, 3.17)**	**0.02**
	T4	**3.33 (2.34, 4.74)**	**<0.01**	**4.07 (2.36, 7.02)**	**<0.01**
AJCC N	N1 (ref)				
	N2	**1.89 (1.71, 2.09)**	**<0.01**	**2.52 (2.19, 2.91)**	**<0.01**
Grade	Well differentiated (ref)				
	Moderately differentiated	1.17 (0.90, 1.51)	0.25	1.40 (0.92, 2.12)	0.12
	Poorly/undifferentiated	**1.49 (1.14, 1.94)**	**<0.01**	**1.85 (1.21, 2.83)**	**<0.01**
LN	<12 LN removed (ref)				
	≥12 LN removed	**0.72 (0.63, 0.82)**	**<0.01**	**0.72 (0.60, 0.87)**	**<0.01**

Abbreviations: AJCC, American Joint Committee on Cancer; CI, confidence interval; HR, hazard ratio; LN, lymph nodes; Q, quartile; ref, reference.

Bold indicates all the percentages in associated with a significant *p*‐values.

When we further stratified survival analysis by patients' age (66–69 vs. ≥70 years), no statistically significant survival benefit was detected in stage II patients for OS and CSS comparing FLOX/OX and 5‐FU/LV (Appendix Table A2). In stage III patients, regardless of age, FLOX/OX was associated with a significant benefit in OS and CSS compared with 5‐FU/LV. For stage III patients <70 years, capecitabine was also associated with a significant OS benefit.

The multivariable Cox regression analysis results estimated from the stratification on the propensity score were slightly different from the results reported in Table 2A,B for the three types of regimens. After propensity score adjustment, there was no statistically significant difference in three types of regimens in OS and cancer‐specific survival in Stage II patients. Among stage III patients, capecitabine and FLOX/OX were associated with improved OS compared with 5/FU/LV, and FLOX/OX was associated with improved CSS compared with 5/FU/LV (Appendix Table A3).

### 3.3 Association of ER/hospitalization and chemotherapy regimen

ER visits and hospitalizations were common among our cohort with about 29.4% of stage II and 38.2% of stage III patients having at least one ER/hospitalization during the chemotherapy treatment period. The hospitalization rate was 18.8% and 24.8% for stage II and III patients, respectively. Capecitabine had the lowest ER/hospitalization rate for both stage II and III patients (Table 3). ER/hospitalization for 5‐FU/LV and FLOX/OX were similar for stage II patients. In stage III patients, 5‐FU/LV had a higher ER/hospitalization rate than FLOX/OX (48.1% vs. 38.7%, respectively). In stage II patients, compared with 5‐FU/LV, capecitabine patients were 48% less likely to have ER/hospitalization, while FLOX/OX was not associated with increased risk of ER/hospitalization (Table 4). For stage III patients, compared to treatment with 5‐FU/LV, patients who received capecitabine and FLOX/OX were 53% and 19% less likely to have ER/hospitalization, respectively. Other factors associated with lower risk of ER/hospitalization among stage III patients include: being male, age 66–69 years, Asian and other race/ethnicity, living in West SEER regions, no comorbidity, and treatment duration ranging from 3 to 6 months.

**TABLE 3:** Distribution of hospitalization or ER visit by patients' characteristics during adjuvant chemotherapy by stage

		Stage II			Stage III		
		N	(%)	*p*	N	(%)	*p*
Regimen	5Fu/LV	87	**33.0**	**<0.01**	301	**48.0**	**<0.01**
	Capecitabine	65	**19.2**		235	**28.9**	
	FLOX/OX	166	**34.5**		1201	**38.7**	
Diagnosis Year	2007–2009	136	32.6	0.08	568	38.5	0.95
	2010–2012	108	29.5		587	38.1	
	2013–2015	74	24.8		582	38.0	
Race	White	249	30.0	0.46	1353	**38.6**	**<0.01**
	Black	23	31.0		165	**43.0**	
	Hispanic	33	28.9		140	**37.6**	
	Other/Unknown	13	20.6		79	**27.4**	
Age	66–69	90	**23.9**	**<0.01**	442	**34.0**	**<0.01**
	70–80	228	**32.3**		1295	**39.9**	
Gender	Female	182	31.8	0.06	966	**41.4**	**<0.01**
	Male	136	26.7		771	**34.8**	
AJCC N	N1	NA			1107	**36.8**	**<0.01**
	N2	NA			630	**41.0**	
AJCC T	T1	NA			54	31.3	**0.05**
	T2	NA			150	37.9	
	T3	217	29.2	0.83	1166	37.7	
	T4	101	29.8		367	41.6	
Surgical removal of lymph nodes	<12 LN removed	62	28.7	0.79	237	39.1	0.62
	≥12 LN removed	256	29.6		1500	38.1	
Grade	Well differentiated	18	24.6	0.10	82	37.6	0.38
	Moderately differentiated	210	28.1		1125	37.6	
	Poorly/undifferentiated	90	34.4		530	39.8	
Census tract median income	Q1 $0–36,811	102	33.0	0.05	465	**39.6**	**<0.01**
	Q2 $36812–51,033	86	33.1		489	**41.3**	
	Q3 $51.34–71,033	69	26.5		421	**37.4**	
	Q4 > $71,034	61	24.3		362	**34.1**	
Census tract non‐high school graduate percent	Q1 > 22.53%	117	34.8	0.06	489	**38.7**	**<0.01**
	Q2 12.96–22.52%	76	29.1		465	**40.9**	
	Q3 7.06–12.95%	66	26.1		396	**36.9**	
	Q4 0.0–7.05%	59	25.5		387	**36.1**	
Marital status	Married	179	30.3	0.46	922	**36.2**	**<0.01**
	Not married	139	28.3		815	**40.7**	
Region	Northeast	54	29.1	**0.04**	298	**41.1**	**<0.01**
	Midwest	45	40.1		203	**42.3**	
	South	127	29.8		779	**40.3**	
	West	92	25.7		457	**32.4**	
Enrolled in Medicare State‐buy‐in	No	255	29.1	0.69	1421	37.6	0.05
	Yes	63	30.5		316	41.3	
Urban	Big Metro	141	25.7	**0.04**	810	36.2	0.05
	Metro	101	32.8		572	40.7	
	Urban	60	32.0		311	39.2	
	Rural	16	41.0		44	39.6	
Charlson index	0	157	27.6	0.21	823	**35.2**	**<0.01**
	1	86	29.3		437	**37.0**	
	2+	75	34.0		477	**46.3**	
Chemotherapy duration	<90 days	87	33.9	**<0.01**	358	**43.8**	**<0.01**
	90–180 days	139	23.2		851	**31.7**	
	181+ days	92	40.5		528	**50.5**	

Abbreviations: AJCC, American Joint Committee on Cancer; LN, lymph nodes; Q, quartile. NA, not applicable.

Bold indicates all the percentages in associated with a significant *p*‐values.

**TABLE 4:** Logistic regression models for factors associated with hospitalization/ER visits by cancer stage

		Stage II		Stage III	
		OR (95%CI)	*p*	OR (95%CI)	*p*
Regimen	5‐FU/LV	Reference		Reference	
	Capecitabine	**0.52 (0.35, 0.78)**	**<0.01**	**0.47 (0.37, 0.59)**	**<0.01**
	FLOX/OX	1.30 (0.92, 1.83)	0.14	**0.81 (0.67, 0.97)**	**0.02**
Year dx	2007–2009				
	2010–2012	0.84 (0.60, 1.17)	0.31	1.01 (0.86, 1.18)	0.92
	2013–2015	0.78 (0.54, 1.14)	0.20	1.01 (0.86, 1.18)	0.91
Gender	Female	Reference		Reference	
	Male	**0.73 (0.55, 0.97**)	**0.03**	**0.76 (0.67, 0.87)**	**<0.01**
Age	66–69	Reference		Reference	
	70–80	**1.51 (1.11, 2.05)**	**<0.01**	**1.30 (1.13, 1.50)**	**<0.01**
Race	White	Reference		Reference	
	Black	0.99 (0.56, 1.75)	0.98	1.07 (0.84, 1.34)	0.59
	Hispanic	0.78 (0.48, 1.28)	0.33	0.91 (0.72, 1.17)	0.47
	Other/Unknown	0.58 (0.29, 1.17)	0.13	**0.68 (0.51, 0.92)**	**0.01**
Marital	Married				
	Not married	0.80 (0.59, 1.07)	0.13	1.01 (0.88, 1.15)	0.92
Region	Northeast	Reference		Reference	
	Midwest	1.48 (0.86, 2.57)	0.16	1.05 (0.82, 1.36)	0.69
	South	0.86 (0.56, 1.34)	0.52	0.97 (0.80, 1.18)	0.77
	West	0.85 (0.55, 1.33)	0.48	**0.73 (0.59, 0.89)**	**<0.01**
Urban	Big Metro	Reference		Reference	
	Metro	1.51 (1.07, 2.12)	0.02	1.14 (0.99, 1.33)	0.07
	Urban	1.37 (0.89, 2.11)	0.15	0.99 (0.82, 1.20)	0.95
	Rural	1.72 (0.82, 3.60)	0.15	1.03 (0.68, 1.56)	0.88
Statebuyin	No				
	Yes	1.22 (0.82, 1.81)	0.33	**1.29 (1.07, 1.55)**	**<0.01**
Census tract median income	Q1 $0–36,811	Reference		Reference	
	Q2 $36812–51,033	1.22 (0.81, 1.84)	0.34	1.07 (0.89, 1.29)	0.48
	Q3 $51.34–71,033	0.97 (0.60, 1.59)	0.92	0.92 (0.74, 1.15)	0.48
	Q4 > $71,034	1.03 (0.56, 1.87)	0.93	0.85 (0.65, 1.12)	0.25
Census tract non‐HS pct	Q1 > 22.53%	Reference		Reference	
	Q2 12.96–22.52%	0.73 (0.49, 1.08)	0.12	1.10 (0.91, 1.33)	0.31
	Q3 7.06–12.95%	0.63 (0.39, 1.02)	0.06	1.05 (0.84, 1.32)	0.67
	Q4 0.0–7.05%	0.69 (0.39, 1.21)	0.19	1.14 (0.88, 1.47)	0.31
CHARLSON	0				
	1	1.08 (0.77, 1.51)	0.65	1.06 (0.91, 1.23)	0.45
	2+	1.43 (0.99, 2.06)	0.06	**1.61 (1.37, 1.88)**	**<0.01**
Duration	<90 days	Reference		Reference	
	90‐180 days	0.53 (0.37, 0.74)	**<0.01**	**0.59 (0.50, 0.69)**	**<0.01**
	181 + days	1.21 (0.81, 1.80)	0.35	**1.22 (1.00, 1.47)**	**0.05**
Grade	Well differentiate	Reference		Reference	
	Moderately differentiated	1.20 (0.67, 2.16)	0.55	0.94 (0.70, 1.26)	0.68
	Poorly/undifferentiated	1.54 (0.82, 2.89)	0.18	0.96 (0.70, 1.30)	0.79
LN	<12 nodes removed				
	≥12 LN nodes remove	1.12 (0.78, 1.59)	0.54	0.94 (0.78, 1.13)	0.53
AJCCT	T1	Reference		Reference	
	T2			1.36 (0.92, 2.02)	0.13
	T3	ref		1.23 (0.87, 1.74)	0.24
	T4	1.07 (0.79, 1.46)	0.67	1.40 (0.97, 2.02)	0.07
AJCCN	N1			ref	
	N2			**1.15 (1.00, 1.31)**	**0.04**

Abbreviations: AJCC, American Joint Committee on Cancer; CI, confidence interval; LN, lymph nodes; OR, odds ratio; Q, quartile; ref, reference.

Bold indicates all the percentages in associated with a significant *p*‐values.

## 4 DISCUSSION

In our study, about half of stage II and 70% of stage III colon cancer patients received FLOX/OX. FLOX/OX treatment showed about 5% improvement in stage II and about 10% improvement in 5‐year OS and CSS for stage III patients. The OS and CSS benefits of FLOX/OX were retained in stage III patients ≥70 years. Among the three regimens, capecitabine had the lowest ER/hospitalization rate for both stage II and III patients.

We revealed increasing use of capecitabine, decreasing use of 5‐FU/LV, and increasing use of FLOX/OX between 2007 and 2011, followed by decreasing use in 2012–2015. The reduced use of FLOX/OX after 2011 may have resulted from several clinical trials showing no or small survival benefit of adding oxaliplatin to 5‐FU/LV or capecitabine, especially for patients older than 70 years. In 2011, Yothers et al. found improved survival of patients <70 years treated with FLOX/OX but not in those ≥70 years.^8^ McCleary and colleagues analyzed data from seven phase III trials in the Adjuvant Colon Cancer End Points (ACCENT) database.^15^ It showed no improvement in OS or disease‐free survival of oxaliplatin‐related regimens compared with 5‐FU/LV in patients ≥70 years. Several studies indicate that adding oxaliplatin to 5‐FU/LV or capecitabine has limited benefit to patients' survival.^15^

We confirmed the survival benefit of FLOX/OX compared with capecitabine and 5‐FU/LV for all patients regardless of age. We found that patients who received FLOX/OX had 28% lower risk of OS than those who received 5‐FU/LV. This result is consistent with a study by Andre et al. of six‐year OS, where there was a 4% improvement in FOLFOX4 versus 5‐FU/LV for stage III patients.^9^ In 2011, Yothers et al. found 20% reduction in risk of death for OS comparing FLOX versus 5‐FU/LV in stage III patients.^8^ In 2012, Sanoff et al. found a 20% lower risk of death comparing FLOX/OX with non‐FLOX/OX in stage III patients.^16^ We observed about 27% lower risk of death associated with FLOX/OX versus 5‐FU/LV in stage II patients. This result is different from other studies which found no difference in OS for stage II patients.^8^, ^9^, ^17^ The reason for our significant finding may be due to our patients receiving more recent treatment compared to Weiss' study, which included patients treated in 1992–2005 when FLOX/OX was not widely used in stage II patients. Also, Weiss' study compared adjuvant therapy in patients without evaluating details on the types of regimens the patients received, or the duration of chemotherapy treatment.^17^ Compared to Andre et al., our cohort had a longer follow‐up time (9 years vs Andre and colleagues' 6‐year follow‐up).^9^

Treatment cost is associated with the type of adjuvant chemotherapy regimen received because of high prices, possible toxicity, and limited efficacy.^5^ Various chemotherapy regimens are associated with differing costs of treatment. The North American data suggest that lifetime costs of colorectal cancer treatment are close to $100,000.^5^ The cost breakdown of chemotherapy regimens showed 5‐FU/LV costed from $11,846.45 to $54,943.93, capecitabine from $17,439.99 to $29,114.41, and FLOX from $26,666.80 to $78,711.02.^18^, ^19^, ^20^, ^21^, ^22^ Our analysis shows that patients in lower income brackets or patients who are a part of the state‐buy‐in program often receive cheaper regimens (5‐FU/LV, capecitabine) than patients in higher income brackets or who are not in state‐buy‐in program (i.e., they are more likely to receive FLOX/OX).

Another factor that is associated with the chemotherapy regimen received is toxicity. Chemotherapy has both acute side effects (myelosuppression, diarrhea, hand‐foot syndrome) and chronic side effects (peripheral sensory neuropathy).^3^ We found that capecitabine use was associated with significantly less hospitalizations/ER than 5‐FU/LV. We discovered that stage III patients who were part of the state‐buy‐in program (and thus more likely treated with 5‐FU/LV) were also more likely to be hospitalized compared to patients who were not part of it (likely treated with capecitabine or FLOX), which further confirms our findings. A meta‐analysis of clinical trials showed that capecitabine and FLOX had similar toxicities.^23^

Our survival analysis suggests that receiving chemotherapy for 3 to 6 months results in a colon cancer overall and cause‐specific survival benefit. The current standard treatment for stage III patients is 6 months of FLOX or CAPOX.^2^, ^3^ Many studies have demonstrated that 6 months of chemotherapy provides a relatively small, though significant, increase in survival as compared to 3 months. For high‐risk stage II patients, 3 months is more often recommended than 6 months.^24^ Our results demonstrated a survival benefit for any treatment over 3 months, with 6 months having the largest advantage. In addition to regimens and duration of chemotherapy treatment, we found a potentially modifiable factor, surgical removal of ≥12 lymph nodes, that was associated with improved OS and CSS among colon cancer patients regardless of cancer stage. Patients who had ≥12 lymph nodes removed in our study were associated with about 30% less risk of colon cancer‐related death compared with those who had <12 lymph nodes removed. This finding is similar compared with another study by Staegge et al., who found an association between removing of ≥12 lymph nodes and improved overall and relative survival in colon cancer patients.^25^

A major strength of our study is that we used large population‐based datasets with a 9‐year follow‐up period to evaluate the trends and survival outcomes of chemotherapy for stage II and III patients. It provides a real‐world assessment of how 5‐FU/LV, capecitabine, and FLOX/OX were used in patients >65 years, the association of treatment regimen type with survival, and treatment toxicities between 2007 and 2015. One limitation is that claims data lack information on patients' physical function and frailty; these characteristics may influence the choice of chemotherapy regimen received, treatment toxicity, and survival. Second, cancer registry data do not include patients' cancer recurrence or metastasis which could affect patients' survival. Also, there is no information on patients' microsatellite instability (MSI) status which is an important factor in chemotherapy treatment decisions as patients with MSI‐high tumors will likely not receive 5‐FU/LV.^26^ Lastly, our cohort was a relatively “healthy” patient group as they received both colectomy and chemotherapy within 6 months of cancer diagnosis, which may limit the generalizability of our results to the overall colon cancer patients >65 years.

In conclusion, our study addresses the survival benefit and treatment patterns of three adjuvant chemotherapy regimens for stage II and III colon cancer patients. Capecitabine has the lowest toxicities based on hospitalizations/ER. We can conclude that in stage III patients specifically, FLOX/OX has a survival benefit over other three regimens.

## AUTHOR CONTRIBUTIONS

Emily Jones: Investigation, writing original draft, and review. Hui Zhao: Investigation, methodology, data interpretation, and review. Zhigang Duan: Data analysis, visualization, review and editing. Thinh Nguyen: Investigation and review. Sharon H. Giordano: Investigation, data acquisition, and supervision.

## FUNDING INFORMATION

This study was supported in part by the NCI P30 CA016672 and by the Duncan Family Institute.

Dr. Giordano is supported by a Cancer Prevention & Research Institute of Texas grant (RP160674) and Komen SAC150061.

## CONFLICTS OF INTEREST

No conflicts of interest to disclose.

## ETHICS STATEMENT

This study was considered exempt of Institutional review board approval because the data are de‐identified.

## Data Availability

The datasets generated for this study are based on SEER‐Medicare data with cancer cases diagnosed between 2007 and 2015 and claims till December 2016. The data can be purchased through the National Cancer Institute: https://healthcaredelivery.cancer.gov/seermedicare/obtain/cost.html.

## APPENDIX A

**TABLE A1:** International Classification of Diseases 9th or 10th Revision and Healthcare Common Procedure Coding System codes used to define colon cancer surgery, chemotherapy, and emergency room visit

Surgery	ICD‐9/10 CM codes: 458,0DTE,0DTF,0DTG,0DTK,0DTL,0DTM,0DTN,457,0DBE,0DBF,0DBG,0DBK,0DBL,0DBM,0DBN HCPCS codes: 44139,44,140,44,141,44,143,44,145,44,146,44,147,44,150,44,151,44,155,44,156,44,157,44,158,44,160, 44,204,44,205,44,206,44,207,44,208,44,210,44,211,44,212,44,213
Chemotherapy	HCPCS codes: J9190(fluorouracil), J8521(capecitabine), J0640(leucovorin), J9263(oxaliplatin)
Emergency Room Visit	HCPCS codes: 99281,99,282,99,283,99,284,99,285 Revenue center codes: 0450–0459, 0981
National Drug Code Numbers	Capecitabine 00004110020, 00004110116, 00004110150, 00004110175, 00093747306, 00093747489, 00378251191, 00378251278, 16,729,007,212, 16,729,007,329, 42,291,019,112, 51,079,051,005, 54,868,414,300, 54,868,414,303, 54,868,526,002 Leucovorin 00054449613, 00054449625, 00054449705, 00054449710, 00054449810, 00054449911, 00555048401, 00555048402, 00555048527, 00703514001, 00703514501, 25,021,081,310, 25,021,081,430, 25,021,081,467, 25,021,081,530, 25,021,081,567, 25,021,081,630, 51,079,058,205, 55,390,000,901, 55,390,005,110, 55,390,005,301, 55,390,005,401, 63,323,071,050, 63,323,071,100, 68,152,010,100 Fluorouracil 00023081230, 00066715030, 00187320210, 00187320447, 00187395364, 00187520030, 00378479106, 00703301513, 00703301912, 10,139,006,301, 10,139,006,311, 10,139,006,312, 10,139,006,320, 16,110,081,230, 16,729,027,638, 16,729,027,667, 16,729,027,668, 43,547,025,801, 43,547,025,901, 51,672,406,201, 51,672,406,301, 51,672,411,806, 63,323,011,710, 63,323,011,718, 63,323,011,720, 63,323,011,751, 63,323,011,761, 66,530,024,940, 66,530,025,830, 66,758,004,401, 66,758,004,403, 68,682,000,431 Oxaliplatin 00024059010, 00024059120, 00024059240, 00069007001, 00069007401, 00703398501, 00703398601, 25,021,021,120, 25,021,021,250, 25,021,023,310, 41,616,017,640, 41,616,017,840, 47,335,017,640, 47,335,017,840, 61,703,036,318, 61,703,036,322, 63,323,021,220, 67,457,044,220

Abbreviations: HCPCS, Healthcare Common Procedure Coding System; ICD‐9/10 CM, International Classification of Diseases 9th or 10th Revision.

**TABLE A2:** Multivariable Cox regression analysis of chemotherapy regimen by stage and by age^a^

	Age 66–69 years		Age ≥70 years	
	HR (95% CI)	*p*‐value	HR (95% CI)	*p*‐value
Stage II				
Overall survival				
5‐FU/LV	Reference		Reference	
Capecitabine	1.21 (0.61–2.41)	0.58	0.9 (0.63–1.27)	0.54
FLOX/OX	0.74 (0.38–1.42)	0.36	0.73 (0.52–1.01)	0.06
Cancer‐specific survival				
5‐FU/LV	Reference		Reference	
Capecitabine	0.79 (0.21–2.94)	0.72	1.05 (0.62–1.78)	0.87
FLOX/OX	0.54 (0.16–1.8)	0.32	0.77 (0.47–1.27)	0.31
Stage III				
Overall survival				
5‐FU/LV	Reference		Reference	
Capecitabine	0.54 (0.37–0.81)	<0.01	0.92 (0.77–1.09)	0.39
FLOX/OX	0.49 (0.37–0.65)	<0.01	0.74 (0.64–0.86)	<0.01
Cancer specific survival				
5‐FU/LV	Reference		Reference	
Capecitabine	0.67 (0.38–1.2)	0.18	0.94 (0.72–1.23)	0.66
FLOX/OX	0.53 (0.35–0.81)	<0.01	0.79 (0.64–0.98)	0.03

Abbreviations: CI, confidence interval; HR, hazard ratio.

a Multivariable Cox regression model was adjusted by patients' sociodemographic factors.

**TABLE A3:** Multivariable Cox regression results of chemotherapy regimens with stratification propensity score adjustment

Regimens	Hazard ratio (95% CI)	*p*	Hazard ratio (95% CI)	*p*
	Overall survival		Cancer‐specific survival	
Stage II				
5‐FU/LV (ref)				
Capecitabine	0.98 (0.73, 1.31)	0.90	0.93 (0.60, 1.46)	0.75
FLOX/OX	0.74 (0.53, 1.02)	0.07	0.68 (0.41, 1.12)	0.13
Stage III				
5‐FU/LV (ref)				
Capecitabine	0.80 (0.68, 0.94)	0.01	0.81 (0.64, 1.02)	0.07
FLOX/OX	0.73 (0.58, 0.92)	0.01	0.67 (0.48, 0.93)	0.02
